# Can a Smartphone Application Help Address Barriers to Reporting Substandard/Falsified Medical Products? A Pilot Study in Tanzania and Indonesia

**DOI:** 10.9745/GHSP-D-23-00034

**Published:** 2023-08-28

**Authors:** Janelle M. Wagnild, Diana Lee, Babatunde Jayeola, Penny K. Lukito, Adam Fimbo, Kate Hampshire

**Affiliations:** a Department of Anthropology, Durham University, Durham, United Kingdom.; b World Health Organization, Geneva, Switzerland.; c Indonesia National Agency of Drug and Food Control, Jakarta, Indonesia.; d Tanzania Medicines and Medical Devices Authority, Dodoma, Tanzania.

## Abstract

**Introduction:**

Reporting is an essential component of efforts to combat the distribution and circulation of substandard and falsified (SF) medical products worldwide. However, little is known about why health care professionals (HCPs) do not report suspect products to the national medicine regulatory authority (NMRA) and what measures might address this. This pilot study aimed to assess the utility of a smartphone application for reporting SF medical products in Tanzania and Indonesia.

**Methods:**

At baseline, in 2017, HCPs completed a survey describing perceived barriers to reporting and received training in the identification of SF products and received use of the smartphone reporting application (N=309). The application reporting system was piloted for 6 months. Evaluations took place with HCPs and NMRA staff at the midpoint and endline of the pilot study (2018).

**Results:**

At baseline, HCPs surveyed (n=254) identified the following key barriers to reporting: difficulties identifying SF products, frustrations with existing reporting systems, and fears that reporting may have personal or reputational repercussions. During the pilot period, HCPs submitted a total of 36 reports of 27 products to the NMRAs in their respective countries; of these, 8 products were determined to be SF and 2 were unregistered. In all 10 cases, appropriate regulatory action was taken. Feedback from HCPs and NMRA staff was positive in both countries, suggesting that the application addressed several barriers to reporting as it was convenient and, importantly, opened a line of communication between HCPs and the NMRA. However, the application did not address all barriers to reporting, such as concerns of repercussions.

**Conclusion:**

The findings suggest that this smartphone application may be useful for improving HCPs’ reporting of suspected SF products. Developing and piloting similar reporting applications in other countries and contexts is required.

## Abstract (precis)

A training workshop & smartphone app for health care professionals to report suspected substandard or falsified medical products was found to be effective and more convenient than existing reporting systems.

## Teaser

**Key Findings:**

- Training and roll-out of a smartphone application for reporting suspected substandard or falsified medical products by health care professionals was effective, resulting in the recall of 8 medical products in a 6-month pilot period.
- The convenience of the smartphone application made it easier for health care professionals to report suspect products, but some barriers to reporting, such as fears of repercussions, were not addressed by the application.
- This study confirms the importance of 3 prerequisites for effective reporting: ability to identify suspect products, easy access to appropriate reporting tools, and protection from possible reprisals or other repercussions.

**Key Implications:**

- National stakeholders should pilot similar smartphone applications for reporting substandard and falsified medical products within their own contexts.
- A key to this pilot’s success was the development of standard operating procedures for reporting with the national medicine regulatory authorities before the application roll-out. Future applications should fit within existing standard operating procedures for reporting wherever possible.

## INTRODUCTION

### The Global Problem of Substandard and Falsified Medical Products

Substandard and falsified (SF) medical products pose a major threat to public health and socioeconomic development, particularly in low- and middle-income countries (LMICs), where regulatory capacity tends to be weaker and supply chains more difficult to control.^1^^–^^3^ In terminology agreed upon by the World Health Assembly,^3^ substandard medical products have been authorized by national authorities but fail to meet either quality standards or specifications, or both. “Falsification” refers to the deliberate (fraudulent) misrepresentation of a drug’s identity, composition, or source. A further concern is “unregistered/unlicensed” products, which have not been evaluated and/or approved by the relevant national or regional regulatory authority.^2^^,^^4^

In a landmark study published in 2017, the World Health Organization (WHO) estimated that more than 10% of pharmaceutical products sold in LMICs are likely to be SF, resulting directly in an estimated 200,000 child deaths each year from malaria and pneumonia alone.^1^ More recent research confirms that the problem persists despite ongoing regulatory efforts, causing enormous human suffering and contributing significantly to antimicrobial resistance.^5^^–^^8^ An estimated US$30.5 billion is spent each year on SF medical products in LMICs,^3^ with the poorest and most vulnerable populations shouldering a disproportionate burden.^9^^,^^10^ Experience of ineffective treatment may also undermine trust in formal health systems, perhaps pushing patients toward unregulated sources.^1^^,^^2^^,^^11^

More than 10% of pharmaceutical products sold in LMICs are estimated to be substandard or falsified, resulting directly in an estimated 200,000 child deaths each year from malaria and pneumonia alone.

### Global Surveillance and Barriers to Reporting

Effective monitoring and surveillance are widely regarded as crucial to the fight against SF medical products. In 2012, WHO launched its flagship Global Surveillance and Monitoring System, with the aim of “work[ing] with WHO Member States to improve the quality of reporting of substandard and falsified medical products.”^2^ The system receives, collates, and responds to reports from national medicine regulatory authorities (NMRAs) and associated organizations. However, NMRAs rely heavily on the willingness and ability of those working “on the ground” to identify and report suspect products.^11^^,^^12^

Very little is currently known about reporting behaviors of health care professionals (HCPs) operating at the “grassroots” level, a significant gap given their importance to the system as a whole. WHO acknowledged this in a recent evaluation of the Global Surveillance and Monitoring System^2^:

*Health care professionals are a source of accurate and often reliable reports but … a number of factors may also lead to a culture of non-reporting. Barriers identified include a lack of awareness, either no system or no method for reporting, overcomplicated reporting systems, low response from regulatory authorities, or a lack of feedback . . . . Worryingly, health care professionals sometimes cite a fear of reprisals either from their managers or those engaged in distributing substandard and falsified medical products. A fear of corruption and a concern that they may be open to prosecution or civil actions themselves may discourage health care professionals from reporting suspect products. These are more difficult issues to confront, but failure to address them will cause the problem of underreporting to remain.*

This pilot study responds directly to this gap. Reporting on the development and deployment of a dedicated smartphone reporting application for HCPs in 2 LMICs, Tanzania and Indonesia, this study addresses 2 key questions:

1. What are the main barriers to HCPs reporting suspected SF medical products in Tanzania and Indonesia?
2. How effectively can a dedicated smartphone application help overcome these barriers?

## METHODS

### Setting and Participants

The pilot study was conducted in Tanzania and Indonesia in 2017–2018, led by WHO, in coordination with the Member State Mechanism,^13^ in partnership with NMRAs in both countries: the Tanzanian Medicines and Medical Devices Authority (TMDA) and National Agency of Drug and Food Control (BPOM) in Indonesia. Before conducting this study, both countries had mainly paper-based mechanisms in place for HCPs to report adverse reactions and product quality concerns, with some additional electronic reporting mechanisms available, including resources on NMRA websites, reporting hotlines and emails, and nondedicated smartphone applications (TMDA ADR Reporting Tool in Tanzania; HALO BPOM in Indonesia). However, these have remained underused.

The intervention and evaluation were delivered in 7 regions in Tanzania (Arusha, Dar es Salaam, Dodoma, Mbeya, Mtwara, Mwanza, and Tabora) and 6 provinces in Indonesia (Jawa Tengah, Jawa Timur, Banten, Jawa Barat, DI Yogyakarta, and DKI Jakarta). Study participants were HCPs (including physicians, pharmacists, nurses, dispensers, and technicians) working at public and private hospitals, public and private health centers, and a very small number (n=3, Tanzania only) at private dispensaries. In Tanzania, 180 HCPs were purposively recruited from a total of 59 health facilities; in Indonesia, 129 HCPs were purposively recruited from 62 facilities. Characteristics of these health facilities are shown in Table 1. To participate in the study, HCPs were required to have a smartphone and to be proficient in the working language of the study (English in Tanzania; Bahasa Indonesian in Indonesia).

**TABLE 1.** Characteristics of Health Facilities in Pilot Study on Smartphone Application to Report Substandard or Falsified Medical Products

	**Tanzania, %** (n=59)	**Indonesia, % ** (n=62)
**Location**		
Urban	78	64
Rural	22	36
**Sector**		
Public	57	41
Private	41	56
Other/unknown	2	3
**Type**		
Hospital	76	85
Primary health care facility	24	15

### The Smartphone Application

The smartphone application was developed by Crimson Tide (United Kingdom) based on their multipurpose mpro5 application in collaboration with the WHO and NMRAs. The app was designed to be quick and simple to use, enabling HCPs to complete and submit a report to NMRAs in less than 90 seconds with minimal manual data entry. Reporters uploaded photographs of the suspect product, including barcodes where applicable, and responded to 2 multiple-choice questions: (1) was the product suspected to be substandard or falsified? and (2) did the reporter suspect that the product had caused an adverse reaction or unexpected lack of efficacy? When submitted, the report (as a PDF) was automatically sent to the respective NMRA local and national focal points for further follow-up. Initially, the app included an additional back-end system through which follow-up actions could be scheduled and assigned to NMRA staff. However, this was abandoned mid-pilot due to usability issues, and reports were managed manually using pre-pilot methods (i.e., Excel).

The smartphone app was designed to be quick and simple to use, enabling HCPs to complete and submit a report to NMRAs in less than 90 seconds with minimal manual data entry.

To maximize its usability, the app was designed to work with multiple operating systems (including iOS, Android, and Windows) with compatibility across device types (including older smartphone models) and was available in different languages. It could be used offline (in recognition of network coverage limitations), with reports sending automatically once a connection was established. Draft reports could be saved for later completion using an outbox function. Submitted reports were not saved locally on the phone, thus minimizing storage space requirements.

### Study Procedures

#### Development Workshops

In both countries, initial workshops were held with representatives from WHO; national and regional focal points from NMRAs; local pharmacovigilance, laboratory, inspection, information technology, and enforcement teams; and the application developer. Existing NMRA reporting policies and procedures for SF products were reviewed and adapted, and standard operating procedures were finalized for each country, with “closing the feedback loop” (sharing incident outcomes with HCPs) identified as an essential component.

#### Training, Baseline Survey, and ApplicationRoll-Out

Regional NMRA staff (“local leads”) then held training workshops for HCPs, covering identification of SF medical products (using recent country-specific examples) and use of the application (how to take good photos and ensure essential information was captured) and providing relevant support and advocacy materials. All attendees were invited to complete an anonymous baseline survey (July–August 2017) describing any previous training and reporting of SF medical products, as well as perceived barriers to reporting. Responses were available for n=155 (86%) in Tanzania and n=99 (77%) in Indonesia. After this training, the application was deployed for 6 months, with HCPs able to begin reporting immediately.

#### Evaluation

Midpoint and endpoint evaluations were conducted during the 6-month deployment period. At midpoint, WHO and local and national leads visited participating health facilities in 3 regions per country to solicit HCPs’ feedback on the application and the training they had received. Endpoint evaluation meetings were held with WHO and NMRA representatives (national focal points and local leads) to review reports submitted by HCPs, collate experiences, and discuss lessons learned.

Further detail on any of the study procedures is available on request.

### Analysis

Survey responses were tabulated, and free responses describing barriers to reporting were grouped thematically. Reports submitted during the application deployment period were also tabulated and summarized.

### Ethical Approval

Before the study, senior leadership approval from the heads of the NMRAs was sought, and close communication between the NMRAs and WHO was maintained throughout the course of the pilot study. Ethical approval for secondary analysis of this dataset was granted by Durham University Anthropology Ethics Committee.

## RESULTS

### Baseline Barriers to Reporting

The vast majority of HCPs in both countries had never previously received training on SF medicines (80% in Tanzania, 82% in Indonesia). Only 24% of HCPs in Tanzania and just 1% in Indonesia had ever personally reported a suspected SF medical product before the study. In both countries, the majority of HCPs (72% in Tanzania and 61% in Indonesia) identified various hindrances to reporting (Table 2). These were generally similar between the countries, albeit with some differences in frequency of reporting and how those barriers were experienced.

**TABLE 2.** Reported Barriers to Reporting Substandard and Falsified Medical Products Among Health Care Professionals in Tanzania and Indonesia

**Theme**	**Subthemes**	**Example Quotations, Tanzania**	**Example Quotations, Indonesia**
Difficulties in identifying substandard or falsified products	Difficulty identifying substandard and falsified products	“Knowledge among the health workers on how to identify and report counterfeit drugs/medical products.”	“To judge whether it is a fake/substandard drug or not is difficult.” “Not knowing/not being able to distinguish/recognize real/fake drugs.” “There is a fear that the report we make is wrong.”
	Lack of general awareness/knowledge about substandard and falsified products	“People are not knowledgeable on substandard and falsified medical products.”	“If no one reports to us if there is a drug that is suspected to be fake/illegal.”
Technical and logistical frustrations with current reporting systems	Current reporting process is unwieldy	“Complicated yellow form for filling substandard and falsified medical product.” “The system we use is not user-friendly - it is time consuming.”	“There is no way to quickly report drugs.” “Worry/fear the process becomes long/burdensome.” “There is no easier way of reporting yet.”
	Time/capacity constraints on reporting	“Time limitations.” “Short staff.”	“Dense work at the hospital.”
	No (prompt) feedback from national medicine regulatory authority after reporting	“Lack of quick feedback mechanism after reporting.” “Follow-up and feedback of reported cases was not easy. It reduces the morale of reporting.” “Poor feedback from authority.”	“Fear that there is no follow-up from the parties concerned.”
	Health care professional and public do not know how to report	“Health care provider doesn’t have the education on what to do.” “Awareness of the public on how to report the resulted medical effect.”	“Do not know clearly the reporting process.”
Fears of repercussions: personal safety and reputational risks	Risks to personal security	“Threaten to reporters.” “No trust for privacy after reporting the matter.”	“I’m afraid of because of reporting this, the hospital could get into trouble and lose employment.” “The reporter’s security guarantee.” “As an informant, there are considerations that need to be taken regarding the reporting of the drug, in the sense that the reporter is not suspected of more than his role so that legal protection is needed for the reporter.”
	Reputational risks	Not applicable	“When an incident occurs due to substandard or counterfeit drugs in my hospital, then it is reported, it will affect the public perception of my hospital even though the cause is unknown, so it is not reported.” “Barrier/paradigm that hospitals become fake drug carriers.”

#### Difficulties in Identifying SF Products

To report an SF product, first, the HCP has to be able to identify it. SF medicines are typically designed to be near-perfect replicas of the authentic product, making identification challenging, even for trained professionals. HCPs in both countries (especially in Indonesia) said that they lacked the skills and confidence to identify SF products, making it difficult to report them. Some worried that they might inadvertently file a false report, “There is a fear that the report we make is wrong.” Others suggested that low public awareness meant that patients rarely report adverse events, potentially allowing SF products to slip through the net.

HCPs in both countries reported that a barrier to reporting these products was their lack of skills and confidence to identify SF products.

#### Technical and Logistical Frustrations With Current Reporting Systems

The most widely noted barriers to reporting in both countries were difficulties, frustrations, and uncertainties with the existing reporting systems. In both countries, several respondents mentioned not knowing how or to whom to report. This was particularly the case in Indonesia, where knowledge and experience of existing systems were very low, “There is no way to quickly report drugs.” In Tanzania, respondents described the current paper-based system as “burdensome” and “complicated,” requiring several time-consuming steps that were not compatible with their heavy workloads. Given the effort required to submit a report, a lack of prompt (or in some cases any) feedback from the NMRA was mentioned by many as a major disincentive for future reporting in Tanzania. As noted by a respondent, “It reduces the morale of reporting.” In Indonesia, similar concerns were raised but usually in more general terms around “fear that there is no follow-up” rather than specific experience.

#### Fears of Repercussions: Personal Safety and Reputational Risks

In both countries, concerns for personal security and safety were cited as significant barriers to reporting, especially when reports could not be submitted confidentially. Some HCPs feared facing physical retaliation or threats, while others pointed to the need for “legal protection for the reporter” and the risk of losing employment. In Indonesia only, some respondents also highlighted the wider reputational risks for their institutions (and the wider health care system). Reporting of SF medicines might lead to a loss of public confidence and hospitals being seen as “fake drug carriers,” according to a participant.

### Reporting Data

During the 6-month deployment period, a total of 36 reports of 27 medical products (21 medicines covering a range of drug classes, 3 health supplements, and 3 medical devices) were submitted by HCPs using the smartphone application. In Tanzania, 20 reports of 12 products were submitted (9 medicines and 3 medical devices). Of these, 1 was found to be falsified, 4 were substandard, 2 were unregistered, 3 were genuine, and 2 could not be determined (Table 3). In Indonesia, 16 reports of 15 products (12 medicines and 3 health supplements) were submitted. Of these, 3 (all medicines) were found to be substandard, 8 were genuine, and the remaining 4 could not be determined (Table 4). In both countries, action was taken in all instances where a product was deemed substandard, falsified, or unregulated. NMRAs reported SF products to the WHO Global Surveillance and Monitoring System, and appropriate action (i.e., recalls) was taken.

**TABLE 3.** Descriptions of Reports Submitted via the Smartphone Application in Tanzania and Subsequent Outcomes

**Region**	**n**	**Report Type**	**Product**	**Product Type**	**Product Suspected to Be Substandard or Falsified**	**Product Suspected to Have Caused Adverse Reaction or Unexpected Lack of Efficacy**	**Reporting Reason**	**Testing Results**	**Conclusion**	**TMDA Action**	**Reported to WHO**
Mbeya	6	Medicine	Combiart	Antimalarial	Substandard	No (n=5) Yes (n=1)	N/A	Passed Minilab by TLC but failed visual inspection - foreign marks on tablets and no manufacturing date on primary container	Falsified	Recall	Yes
Mbeya	1	Medical device	NeoVac	IV cannula	Falsified	Yes	Not functioning properly	Sterility failure	Substandard	Recall	Yes
Mbeya	1	Medical device	Kit Kath	IV cannula	Falsified	Yes	Not functioning properly	Sterility failure	Substandard	Recall	Yes
Dar es Salaam	1	Medical device	CareStart Malaria	Rapid diagnostic test	Substandard	Yes	Errored/no results; using up to 5 rapid diagnostic test per patient	N/A	Substandard	Recall	Yes
Dar es Salaam	3	Medicine	Diclofenac Injection	Analgesic	Substandard	Yes (n=2) No (n=1)	N/A	N/A	Substandard	Recall	Yes
Arusha	1	Medicine	Adrenaline	Broncho-dilator/cardiac stimulant	Substandard	Yes	N/A	N/A	Unregistered	Recall	N/A
Tabora	2	Medicine	Metronidazole tablets	Antibiotic/antibacterial/ antiprotozoal	Substandard	Yes (n=1) No (n=1)	N/A	N/A	Unregistered	Recall	N/A
Mwanza	1	Medicine	Tranexamic acid tablets (Tranacad)	Antifibrinolytic	Substandard	Yes	N/A	Not tested due to no sample available	No conclusion	N/A	N/A
Mwanza	1	Medical device	Sterile umbilical cord clamp	Umbilical cord clamp	Substandard	Yes	N/A	Not tested due to no sample available	No conclusion	N/A	N/A
Mwanza	1	Medicine	Coram 10	Antihypertensive	Substandard	Yes	N/A	N/A	Genuine	N/A	N/A
Arusha	1	Medicine	D5 infusion injectable solution	Hydrous dextrose	Substandard	Yes	Leakage of product	N/A	Genuine	N/A	N/A
Mtwara	1	Medicine	LARTEM	Antimalarial	Substandard	Yes	ADRs	N/A	Genuine	N/A	N/A

Abbreviations: ADR, adverse drug reaction; N/A, not applicable; TLC, thin-layer chromatography; TMDA, Tanzanian Medicines and Medical Devices Authority; WHO, World Health Organization.

**TABLE 4.** Descriptions of Reports Submitted via the Smartphone Application in Indonesia and Subsequent Outcomes

**Region**	**n**	**Report Type**	**Product**	**Product Type**	**Do You Suspect This Medical Product to be Substandard or Falsified?**	**Do You Suspect the Product May Have Caused an Adverse Reaction or Unexpected Lack of Efficacy?**	**Reporting Reason**	**Testing Results**	**Conclusion**	**BPOM Action**	**Reported to WHO**
DKI Jakarta	1	Medicine	Cetirizine syrup	Antihistamine	Substandard	No	Leakage of bottle	No sample at facility but another product within the same batch was tested and was within specification	Substandard due to facility’s storage issues	Provided storage guidance	N/A
DKI Jakarta	1	Medicine	Paracetamol syrup	Analgesic	Substandard	No	Bottle half-filled with unclear liquid	No sample at facility but another product within the same batch was tested and was within specification	Substandard due to facility’s storage issues	Provided storage guidance	N/A
Jawa Tengah	1	Medicine	Fresofol 1% MCT/LCT (Propofol)	Anesthetic	Substandard	Yes	ADRs, less effective	Tested; within specification	Genuine	Input for inspection	N/A
Jawa Tengah	1	Medicine	Symbicort Turbuhaler (Bedesonide formoterol fumarate)	Corticosteroid	Falsified	No	Different labeling on primary and secondary packaging	No sample at facility but received manufacturer confirmation that the product was genuine	Genuine	Follow-up with manufacturer on availability of retained samples	N/A
Jawa Timur	1	Medicine	Regivell Spinal 0.5% Bupivacaine-Heavy	Anesthetic	Substandard	Yes	ADRs, less effective	No sample at facility but received manufacturer confirmation that the product was genuine	Genuine	N/A	N/A
Jawa Timur	1	Medicine	Regivell Spinal 0.5% Bupivacaine-Heavy (different batch than above)	Anesthetic	Substandard	Yes	ADRs, less effective	No sample at facility but received manufacturer confirmation that the product was genuine	Genuine	N/A	N/A
DI Yogyakarta	1	Medicine	Ranitidine injection	H2-blocker	Substandard	No	Empty ampule	Not tested due to sample unavailability; note another batch of this product was previously reported to BPOM	Substandard	Recall	No
DI Yogyakarta	1	Medicine	Dexamethasone tablets	Corticosteroid	Substandard	No	Half of primary packaging (strip) was empty	Not tested due to sample unavailability (disposed of by HCP)	No conclusion	Input for inspection	N/A
DI Yogyakarta	1	Medicine	Ifison tablets (prednisone)	Corticosteroid	Falsified	No	Contained 1,000 tablets in bottle	Tested; within specification	Genuine; note manufacturers given grace period to switch from 1,000- to 100-tablet containers	N/A	N/A
DI Yogyakarta	1	Medicine	Methyl prednisolone	Corticosteroid	Substandard	Yes	No tablet in 1 part of strip	Tested; within specification	Genuine	Input for inspection	N/A
DI Yogyakarta	1	Medicine	Fludara oral	Anti-cancer	Substandard	No	Different batch number and expiry date on primary and secondary packaging	No, cannot be sampled due to high cost	Genuine; note this product was permitted on a temporary basis	N/A	N/A
Jawa Barat	1	Medicine	Lincomycin Kapsul	Antibiotic	Substandard	No	Suspected leakage	Tested; within specification	Genuine	N/A	N/A
Jawa Barat	1	Other	Provital Plus	Health supplement	Substandard	No	Partially filled strip (missing tablet)	N/A; out of scope of pilot	N/A; out of scope of pilot	N/A	N/A
Jawa Barat	2	Other	Vossecal	Health supplement	Substandard	No (n=1) Yes (n=1)	1 broken tablet	N/A; out of scope of pilot	N/A; out of scope of pilot	N/A	N/A
Jawa Barat	1	Other	Hi-Bone Active	Health supplement	Substandard	No	2 tablets in same packaging	N/A; out of scope of pilot	N/A; out of scope of pilot	N/A	N/A
Banten	0										

Abbreviations: ADR, adverse drug reaction; BPOM, Indonesia National Agency of Drug and Food Control; HCP, health care professional; LCT long chain triglycerides; MCT, medium chain triglycerides; N/A, not applicable; WHO, World Health Organization.

### HCP Perspectives

Overall, HCPs’ feedback on the pilot study and smartphone application in both countries was unanimously positive. HCPs in both countries found the application to be efficient, user-friendly, and convenient. They also valued the more open lines of communication with NMRAs and the emphasis on ensuring a feedback loop. Particularly in Tanzania, HCPs noted the contrast with the previous burdensome paper-based system.

*Trying to find the right blue paper form in the health facility, trying to find time to complete the form and get all the information required. Then trying to get to the post office when it’s open, relying on the mailing infrastructure to hope that it gets to the TMDA. The NMRA reported that it can take up to 6 months for the form to reach them, and in some cases, it has been damaged (water damage, for example). By the time they get back to the reporter, they have forgotten and moved on.* —HCP, evaluation meeting, Tanzania, taken from meeting notes

HCPs in both countries found the application to be efficient, user-friendly, and convenient.

HCPs also commented positively on the training they received on techniques for identifying SF products, particularly the use of pictorial examples and the training and advocacy materials provided. However, some still expressed a lack of confidence in making assessments identifying SF products and suggested the need for regular refresher training. Moreover, the fear of repercussions still persisted: reporting via the smartphone app was not anonymous, and HCPs noted that permission to report had to be sought internally first. There were also some concerns about privacy and confidentiality around communication with NMRAs.

Some HCPs also noted some challenges in using the app. A few found the app to be incompatible with their smartphones, despite having been designed to work on multiple operating systems and devices. Internet access could also be difficult. Reports could be created and stored offline, but an Internet connection was needed for submission, and many HCPs reported having to use personal resources to purchase the requisite phone data. Finally, although communication with NMRAs improved, feedback could still be delayed due to errors or delays within the application (on either end).

### NMRA Perspectives

In both countries, NMRA staff also provided very positive evaluations. In particular, they noted the increased efficiency of reporting using the smartphone app, particularly the use of photographs, compared with paper-based systems. They also valued having better methods of communication with HCPs and noted the utility of providing HCPs with training and awareness around SF products.

However, NMRA staff noted some difficulties. First, the 48-hour timeframe that NMRA staff had to provide feedback on filed reports could be challenging, particularly when errors or delays in the system delayed the process. Following up with HCPs was also difficult when HCPs would not respond to calls or messages. Second, as previously noted, the back-end system was abandoned mid-pilot because of usability issues. Therefore, scheduling and monitoring of follow-up actions was done manually using Excel, adding an extra time burden. While workload within the pilot study was generally felt to be manageable, there was concern that a broader roll-out may be challenging, particularly in light of competing priorities and/or lack of resources.

### Study Limitations

This study was not originally designed as a piece of academic research; as such, the dataset suffers from several limitations. Most significantly, a lack of reliable baseline data on reporting means we cannot compare pre- and post-pilot reporting levels objectively; we are reliant on the subjective accounts and experiences of the study participants. Second, not all participating HCPs were involved in the midpoint and endpoint evaluations, thus limiting the range of experiences captured. Finally, the study was confined to a small number of regions in 2 countries, so the findings cannot be translated straightforwardly to other contexts.

## DISCUSSION

To our knowledge, this study represents the most substantial dataset available to date on barriers to reporting SF medicines, confirming the importance of 3 key prerequisites for effective reporting: ability to identify suspect products, easy access to appropriate reporting tools, and protection from possible reprisals or other repercussions.^2^^,^^12^

The smartphone app developed and piloted in this study represents an important step toward achieving easy access to appropriate reporting tools by providing an efficient and user-friendly means of reporting suspect products. However, some further technical refinements are needed. The associated training for HCPs around identification of SF products goes some way to addressing the ability to identify suspect products, but as previously mentioned, such products are typically designed to evade easy identification. Open lines of communication with NMRAs may help give HCPs the confidence to report without fear of reprisals if they “get it wrong.” Assuring anonymity and confidentiality will also be crucial—something that was not addressed effectively with the smartphone app in its current form.

The smartphone app developed and piloted in this study represents an important step toward achieving easy access to appropriate reporting tools by providing an efficient and user-friendly means of reporting suspect products.

Finally, this evaluation has highlighted the importance of understanding the interplay between individuals, existing systems, and new technologies and the importance of not assuming that technological innovation alone is enough. For example, NMRAs will need to have sufficient resources and may need to adjust their operating procedures to ensure a rapid and effective response to a projected increase in SF reporting. It will also be important to ensure that the time and cost burdens of reporting do not fall inequitably on rural health workers, who have limited Internet access and financial resources for covering mobile data costs.^14^^,^^15^

## CONCLUSION AND NEXT STEPS

Drawing on our experiences and findings, we propose 3 next steps. First, following further refinement of the app to address technical glitches, formal intervention studies should be conducted in both countries, with larger and more representative groups of HCPs, reliable baseline data, appropriate controls, and sufficient statistical power to detect changes in reporting levels and accuracy. This would provide a solid evidence base on which to scale up.

Second, pending successful intervention studies and roll-out in Tanzania and Indonesia, the applications could be adapted for use in other countries and contexts. To this end, it would be useful to develop user resources (handbooks/web-based) to allow the sharing of lessons learned and best practices between countries, highlighting the importance of factoring in possible resources and other implications for the ways that NMRAs and HCPs operate.

Third, consideration should be given to extending the interventions beyond HCPs and focusing on pharmacies and over-the-counter medicine outlets, often the first point of contact for many people in rural areas who lack access to basic primary care. Community pharmacists and medicine retailers usually have detailed knowledge of the communities they serve and represent a valuable and underused resource in reporting SF medical products.

Finally, a note of caution: technological innovations like the smartphone app are not “magic bullets.” They are a useful part of the arsenal but will only be effective in conjunction with wider-reaching reforms to address higher-level vulnerabilities in pharmaceutical supply chains in Africa and expand access to quality-assured health services.^2^

## Notes

**Peer Reviewed**

**First published online:** August 3, 2023.
